# Vision function in children 10 years after grade 3 or 4 intraventricular haemorrhage with ventricular dilation: A masked prospective study

**DOI:** 10.1111/dmcn.15294

**Published:** 2022-06-23

**Authors:** Cathy Williams, Penny Warnes, Sally Jary, Grace Young, Peter S. Blair, Christopher P. Benton, Helen Miller, Andrew Whitelaw, Ian Pople, Karen Luyt, DRIFT10 study group, Kristian Aquilina, Charlotte Lea, David Odd, Adam Smith‐Collins, William Hollingworth

**Affiliations:** ^1^ Centre for Academic Child Health, Bristol Medical School University of Bristol Bristol UK; ^2^ Bristol Eye Hospital University Hospitals Bristol NHS Foundation Trust Bristol UK; ^3^ Neonatal Neurology Bristol Medical School Bristol UK; ^4^ Bristol Randomised Trials Collaboration Bristol Medical School Bristol UK; ^5^ School of Psychological Science University of Bristol Bristol UK; ^6^ Department of Neurosurgery Bristol Royal Hospital for Children Bristol UK

## Abstract

**Aim:**

We examined children 10 to 11 years after grade 3 or 4 intraventricular haemorrhage and ventricular dilation (IVHVD) and investigated whether the grade of IVHVD affected their visual outcome. We explored associations between visual outcomes with cognitive outcomes and extra support at school.

**Method:**

The visual examinations were part of a 10‐year follow‐up study for children in a randomized trial. Testers followed a protocol and were masked to whether the child had experienced grade 3 or grade 4 IVHVD and all other data.

**Results:**

Thirty‐two children were tested: 24 were male and mean (standard deviation) age was 10 years 5 months (1 year 2 months); range 8 years 9 months to 12 years 9 months. All had at least one visual impairment. The median (interquartile range) number of impairments per child was six (six to nine) for children who experienced a grade 4 IVHVD compared with three (two to four) for children who experienced a grade 3 IVHVD (*p* = 0.003). Each extra vision impairment per child was associated with increased educational support at school, after adjustment for developmental age equivalence (odds ratio = 1.7 [95% confidence interval 1.1–2.6], *p* = 0.015).

**Interpretation:**

Children who experience grade 3 or 4 IVHVD have a high level of visual morbidity at age 10 to 11 years. These children may have unmet visual needs and their outcomes might improve if these needs could be addressed.

**What this paper adds:**

- Parent‐reported questionnaire responses underestimated directly assessed visual morbidity.
- Grade 4 intraventricular haemorrhage and ventricular dilatation (IVHVD) was followed by more vision impairments than grade 3 IVHVD.
- Simple tests of visual perceptual skills correlated with the neuropsychology tests.
- Children with supranuclear eye movement disorders were more likely to be receiving extra help at school.
- Each additional visual impairment increased the likelihood of extra educational support.

## What this paper adds

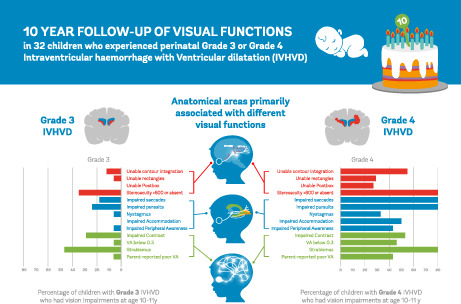

This article's abstract has been translated into Spanish and Portuguese.

Follow the links from the abstract to view the translations.

## ResumenFunción de la visión en niños 10 años después de una hemorragia intraventricular de grado 3 o 4 con dilatación ventricular: un estudio prospectivo enmascarado

**Objetivo:**

Examinamos a niños de 10 a 11 años después de hemorragia intraventricular y dilatación ventricular (IVHVD) de grado 3 o 4 e investigamos si el grado de IVHVD afectó su resultado visual. Exploramos las asociaciones entre los resultados visuales con los resultados cognitivos y el apoyo adicional en la escuela.

**Método:**

Los exámenes visuales fueron parte de un estudio de seguimiento de 10 años para niños en un ensayo aleatorizado. Los evaluadores siguieron un protocolo y se enmascararon si el niño había experimentado IVHVD de grado 3 o grado 4 y todos los demás datos.

**Resultados:**

Treinta y dos niños fueron evaluados: 24 eran varones y la edad media (desviación estándar) era de 10 años 5 meses (1 año 2 meses); rango 8 años 9 meses a 12 años 9 meses. Todos tenían al menos una discapacidad visual. La mediana (rango intercuartílico) del número de deficiencias por niño fue seis (seis a nueve) para los niños que experimentaron IVHVD de grado 4 en comparación con tres (dos a cuatro) para los niños que experimentaron IVHVD de grado 3 (p = 0,003). Cada deficiencia visual adicional por niño se asoció con un mayor apoyo educativo en la escuela, después del ajuste por equivalencia de edad de desarrollo (odds ratio = 1,7 [intervalo de confianza del 95 %: 1,1–2,6], p = 0,015).

**Interpretación:**

Los niños que experimentan IVHVD de grado 3 o 4 tienen un alto nivel de morbilidad visual entre los 10 y los 11 años. Estos niños pueden tener necesidades visuales insatisfechas y sus resultados podrían mejorar si estas necesidades pudieran abordarse.

## RESUMO

**Objetivo:**

Examinamos crianças de 10 a 11 anos após hemorragia intraventricular de grau 3 ou 4 e dilatação ventricular (HIVDV) e investigamos se o grau de HIVDV afetou seu resultado visual. Exploramos associações entre resultados visuais com resultados cognitivos e apoio extra na escola.

**Métodos:**

Os exames visuais fizeram parte de um estudo de acompanhamento de 10 anos para crianças em um estudo randomizado. Os testadores seguiram um protocolo e foram mascarados para saber se a criança havia experimentado HIVDV grau 3 ou grau 4 e todos os outros dados.

**Resultados:**

Trinta e duas crianças foram testadas: 24 eram do sexo masculino e a idade média (desvio padrão) foi de 10 anos 5 meses (1 ano 2 meses); intervalo 8 anos 9 meses a 12 anos 9 meses. Todos tinham pelo menos uma deficiência visual. A mediana (intervalo interquartil) do número de deficiências por criança foi de seis (seis a nove) para crianças que experimentaram uma HIVDV grau 4 em comparação com três (dois a quatro) para crianças que experimentaram uma HIVDV grau 3 (p = 0,003). Cada deficiência visual extra por criança foi associada ao aumento do suporte educacional na escola, após ajuste para equivalência de idade de desenvolvimento (odds ratio = 1,7 [intervalo de confiança de 95% 1,1‐2,6], p = 0,015).

**Interpretação:**

As crianças que sofrem de HIVDV grau 3 ou 4 têm um alto nível de morbidade visual na idade de 10 a 11 anos. Essas crianças podem ter necessidades visuais não atendidas e seus resultados podem melhorar se essas necessidades puderem ser direcionadas.

**Abbreviations**
BAS: British Ability Scales, Third Edition
DAE: developmental age equivalent
DRIFT: drainage, irrigation, and fibrinolytic therapy
IVH: intraventricular haemorrhage
IVHVD: intraventricular haemorrhage and ventricular dilatation
SEN: special educational need

Visual impairments after preterm birth include a higher prevalence of misaligned eyes (strabismus), and refractive errors as well as impairments of visual acuity, visual fields, contrast sensitivity, near‐focusing (accommodation), and vision processing.^1^, ^2^, ^3^, ^4^, ^5^

Intraventricular haemorrhage (IVH) is a common complication of preterm birth and graded 1 to 4 according to severity. Intraventricular haemorrhage and ventricular dilatation (IVHVD) is common and may result in post‐haemorrhagic hydrocephalus requiring the insertion of a drainage device such as a ventriculo‐peritoneal shunt. Some studies report only a few deficits by school age after IVHVD^6^ and some report multiple deficits,^7^ with severity of IVH and the need for neurosurgery being predictors of worse outcome.

We tested visual function in children who were enrolled at birth into a randomized trial of a new treatment for IVHVD called drainage, irrigation, and fibrinolytic therapy (DRIFT). The DRIFT intervention involved elective irrigation of the ventricles after IVHVD and this treatment reduced the prevalence of cognitive impairments at the age of 2 years, in a randomized trial.^8^ The trial children were assessed again at the age of 10 to 11 years when the cognitive benefits associated with receiving DRIFT were found to be sustained.^9^, ^10^ Specifically, the follow‐up study found that survival without severe cognitive disability was 66% in the DRIFT arm compared with 35% in the usual‐care arm (adjusted *p* = 0.003). Secondary outcomes of the follow‐up study included parent‐reported vision, which was ‘good’ (normal or corrects to normal with glasses) in 85% of the children in the DRIFT arm compared with 71% of children in the usual‐care arm (*p* = 0.132). The children in the DRIFT arm also had slightly lower mean scores in a parent‐reported questionnaire designed to elicit responses suggestive of cerebral visual impairment:^11^ DRIFT arm 4.50 versus standard care arm 4.65 (*p* = 0.502). For reasons of space, we did not include the results of direct vision testing in the main paper, so we present them here.

**FIGURE 1:**
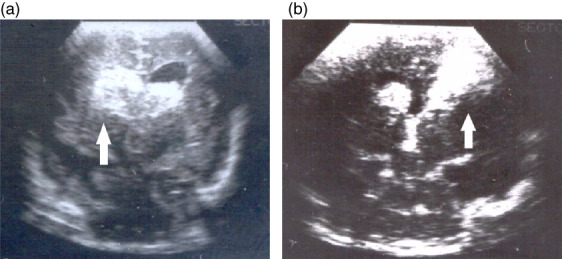
Grades of intraventricular haemorrhage. (a) Grade 3 intraventricular haemorrhage (IVH). Coronal view ultrasound scan showing the lateral ventricles (black) are greatly enlarged and full of clotted blood (white and arrowed). (b) Grade 4 IVH. Coronal view. In addition to intraventricular blood there is a white area of haemorrhagic infarction (white and arrowed).

The follow‐up study was designed and powered to detect reduced cognitive abilities and the visual outcomes were secondary. We thereforepresent the vision findings as exploratory investigations of visual function in children (1) in the two arms of the trial and (2) who experienced grade 4 IVHVD compared with grade 3 (Figure 1). We also compared results obtained using our simple visuoperceptual/visuocognitive tests with the results of the standard neuropsychology tests as a guide to interpretation of the former. Finally, we explored whether having visual impairments was associated with needing extra help at school, as a guide to rehabilitation after IVHVD.

## METHOD

### Approvals, participants, and setting

All 48 survivors from the DRIFT trial in the UK were invited to participate in the 10‐year study. Thirty‐seven were able to attend the research centre in Bristol. Visual assessments were timetabled last in the day and five children were unable to stay because of transport arrangements or tiredness. Testing took place in 2015 to 2016 and the main DRIFT results paper was published in 2020,^10^ paving the way for these vision results to be presented.

Ethical approval was granted by the NHS Health Research Authority, NRES Committee South West ‐ Central Bristol (14/SW/1078). Parents gave written informed consent. The children gave their assent for follow‐up.

### Outcome measures

#### Vision tests

The children were examined by an experienced orthoptist (PW) or ophthalmologist (CW), following a protocol and unaware of the child's history or other results. Testing lasted 40 to 50 minutes per child. The protocol comprised monocular and binocular logarithms of minimum angle of resolution of visual acuity with glasses if worn, or the most advanced acuity test of which the child was capable; cover tests to identify strabismus; observation of nystagmus; accuracy of supranuclear eye movements (pursuits and saccades) using a semi‐quantitative 1 to 5 scale;^12^ dynamic retinoscopy to assess accommodation (near‐focusing); and three simple visuoperceptual/visuocognitive tasks. These last three tests were contour integration cards (where contour integration involves combining elements of information across the visual field into a meaningful whole);^13^ the Lea postbox task; and the Lea rectangles task. We chose these tests because we knew from past experience in our local clinic that they would be accessible for many of the children with developmental problems and because we had normative data to guide interpretation of the results.^14^

We performed non‐cycloplegic autorefraction with a Canon R50 autorefractor (Clement Clarke, Haag Streit UK, Harlow, UK) as a back‐up to the optometric records where available. Children were also offered the opportunity to engage with a locally designed pilot motion‐detection computer game involving sensitivity to coherent motion in a random dot display. With permission, we also reviewed past ophthalmology, optometry, and school medical notes.

As reported in our previous paper and summarized above, the parents were asked about their children's vision using a published inventory^11^ and they were asked to categorize their child's vision using one of four options: ‘normal’; ‘normal with correction’; ‘useful but not fully correctable’; or ‘blind or perceives light only’. For analysis the first two categories were combined as ‘normal with or without correction’ and the last two categories were combined as ‘useful but not correctable to normal, or blind or perceives light only’.

The published inventory questions are given in Appendix S1.

### Additional data

An experienced neuropsychologist administered the British Ability Scales, Third Edition (BAS) test, either for early years or for 4 years upwards, depending on the child's abilities. The published norms were used to derive centiles for the child's abilities. We included the results for the ‘spatial ability’ cluster as these require the use of vision and the ‘verbal ability’ cluster as these do not. We also included a derived ‘developmental age equivalent’ (DAE), calculated by the neuropsychologist on the basis of the data for all the cognitive results for each child, as in the main DRIFT papers.^9^, ^10^

Perinatal data were obtained from original study records and hospital notes on weight and gestation at birth, grade of IVH, and presence of a ventriculo‐peritoneal shunt. Parents were asked about epilepsy, hearing or vision problems, and special educational needs (SENs).

### Analysis

All vision test results were classified as normal or abnormal, using previously reported norms or clinical convention. We derived 12 separate visual impairments, as listed in Table 1. We used medians and interquartile ranges (IQR) to describe the data, and χ^2^ or Fisher's exact tests to compare proportions. We used Spearman's correlation coefficient to examine correlations between continuous variables and a two‐sample Kolmogorov–Smirnov test to explore associations between the distributions of vision test results and the cognitive scores. We examined associations between whether the child was in the top quartile of children according to the number of visual impairments that were detected with the study arm; grade of IVHVD; or with having a SEN; all using logistic regression and we report odds ratios (ORs) with 95% confidence intervals (CIs). Model fit was assessed with a log likelihood ratio χ^2^ statistic. Analyses were done in Stata MP 15.1 (Stata Statistical Software: Release 15, StataCorp LLC., College Station, TX, USA). We did not impute missing data. Where appropriate, we used the variables specified in the main study (birthweight, grade of IVHVD, sex) to adjust for imbalances between the trial arms.

**TABLE 1:** Clinical characteristics and vision impairments on examination at age 10 to 11 years, for 32 children with previous IVHVD

Characteristic	*n* (%) or median (IQR)
Male	24 (75)
Gestation at birth, weeks	27.5 (26.0–29.0)
Birthweight, g	1066.0 (869.0–1394.0)
Grade 4 intraventricular haemorrhage	15 (47)
Insertion of ventriculo‐peritoneal shunt	13 (41)
Epilepsy	4 (12)
Cerebral palsy	17 (53)
Treated for retinopathy of prematurity	4 (13)
Has a special educational need	16 (50)
Attended a special school in previous 12 months	6 (19)
In DRIFT (drainage, irrigation, and fibrinolytic therapy) group	19 (59)
Parent‐reported data: deaf (needs aid or is uncorrectable)	4 (12)
Parent‐reported data: ‘blind’ or ‘useful but not fully correctable’	8 (25)
BAS test: median spatial ability centile for age at age 10 years	5.0 (0.7–39.5)
BAS test: median verbal ability centile for age at age 10 years	9.0 (1.0–45.0)
Developmental age equivalent at age 10 years, months	89.7 (71.9–115.9)
Visual acuity (binocular) worse than 0.3 logMAR	8 (25)
Has a strabismus	20 (62)
Impaired (worse than 66%) contrast sensitivity	13 (41)
Abnormal peripheral awareness	7 (23)
Nystagmus in primary position	6 (19)
Lag in accommodation	6 (24)
Impaired pursuit eye movements (mean <4)	16 (50)
Impaired saccadic (mean <4)	15 (47)
Unable to detect contours using long‐range facilitation (delta >1.0)	8 (29)
Unable/struggled to do the Lea postbox task	4 (12)
Unable to do rectangle matching	5 (16)
No stereoacuity or > 800 seconds/arc	18 (56)

Abbreviations: BAS, British Ability Scales, Third Edition; IQR, interquartile range; IVHVD, intraventricular haemorrhage and ventricular dilatation; logMAR, logarithm of minimum angle of resolution.

## RESULTS

### Participants and prevalence of visual impairments

Characteristics of the 32 children (24 males; mean [standard deviation] age 10 years 5 months [1 year 2 months]; range 8 years 9 months to 12 years 9 months) who attended for vision testing are shown in Table 1, together with the prevalence of each vision impairment. Compared with the rest of the DRIFT participants for whom we had parent‐reported data at 10 years (the five who could not stay for vision tests, 11 UK‐resident children who did not come to Bristol, and four who lived outside the UK), the children included here were less likely to have attended a special school in the previous year (19% vs 70%), or to have an SEN (50% vs 90%), or to have cerebral palsy (CP) (53% vs 82%).

All children were born preterm and had a low birthweight. Approximately half (15 out of 32) had a grade 4 IVHVD, approximately half had CP (17 out of 32), and nearly half (13 out of 32) had a ventriculo‐peritoneal shunt. Four children had epilepsy. Four had been treated with laser surgery for retinopathy of prematurity. Of the 17 with CP, 11 had bilateral spastic CP, five had unilateral spastic CP, and one had ataxic CP.^15^

As expected, cognitive impairments and developmental delay were common (Table 1). The median DAE was approximately 90 months (7 years 6 months) and the range was 7 months to 186 months (15 years 6 months), ranging from being developmentally equivalent to an infant, to having abilities in excess of a typically developing 10‐year‐old child. Age‐scaled centile scores were low for both the vision‐dependent spatial ability cluster and the verbal ability cluster.

No child was free of any visual impairment. Out of the 12 possible impairments, the median (IQR) number per child was four (two to seven). The most frequent was strabismus (*n* = 20), followed by reduced stereoacuity (*n* = 18); impaired pursuit (*n* = 16) or saccadic eye movements (*n* = 15); then impaired contrast sensitivity (*n* = 13), while lags of accommodation; impaired peripheral field awareness; reduced visual acuity and visuoperceptual/visuocognitive problems each affected between three and 10 children. Only one of the children treated for retinopathy of prematurity had impaired peripheral fields on confrontation testing but six other children had reduced awareness in all or part of the periphery.

We obtained optometric records and/or autorefraction data (mean spherical equivalent) within the previous 12 months, for 18 children. The mean spherical equivalent for right and left eyes combined was emmetropic (between −0.50 and + 0.50) for five children, hypermetropic (> + 0.50D) for four, and myopic (<−0.50) for four. We were unable to autorefract the other 14 children because of positioning difficulties, machine error, or nystagmus. Of these, two had normal unaided binocular acuity, 10 had been refracted in the past by hospital and/or their own optometrist and never given glasses, and one had worn glasses previously but then abandoned them.

Ten children did the motion‐detection test. The percentage of moving dots that needed to be congruent for the child to identify the direction of motion reliably ranged from 17% (good sensitivity to motion) to 71% (poor sensitivity to motion); the median (IQR) was 44% (25–64).

The high frequency of visual impairments found on direct assessment contrasts with the parent‐reported questionnaire data suggested that 24 children had normal vision with or without glasses (Table 1).

### Number of visual impairments per child in the two study arms

The median (IQR) number of impairments per child was four (two to eight) in the standard care arm and four (two to six) in the DRIFT arm (Kolmogorov–Smirnov *p* = 0.808). In an unadjusted analysis, the DRIFT arm children were as likely to be in the top quartile for number of visual impairments (seven or more) as the standard care arm: OR = 0.33 (95% CI 0.06–1.8, *p* = 0.210). Using the prespecified adjustments (birthweight, sex, and grade of IVHVD) as in the main paper moved this estimate further away from the null: adjusted OR = 0.07 (95% CI 0.007–1.01, *p* = 0.051).

### Comparison of visual outcomes after grade 3 versus grade 4 IVHVD

**FIGURE 2:**
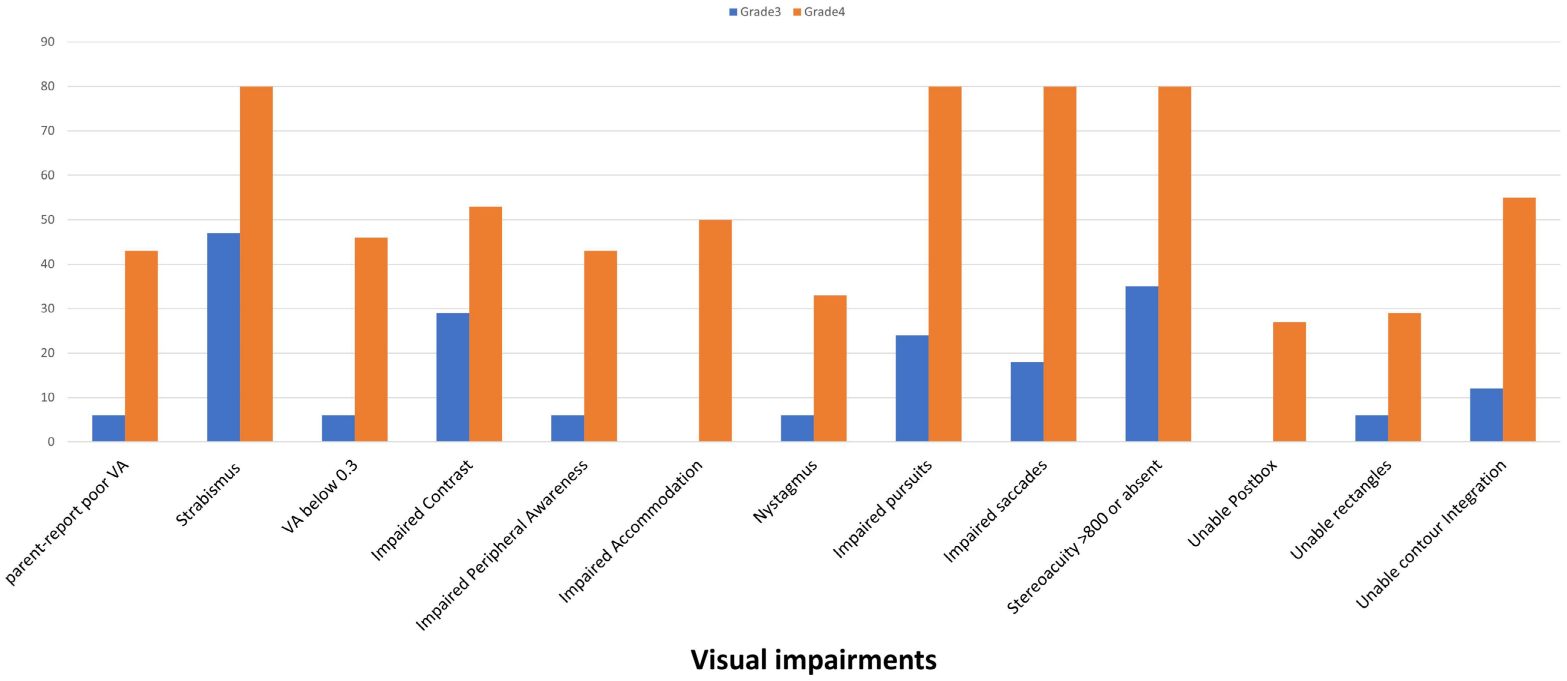
Distribution of visual impairments at age 10 to 11 years for 32 children who as infants suffered ventricular dilatation after grade 3 (*n* = 17) or grade 4 (*n* = 15) intraventricular haemorrhage. One child with grade 4 did not do the rectangles or peripheral awareness tests. Comparisons at *p* < 0.05 using χ^2^ or Fisher's exact tests as appropriate. VA, binocular visual acuity.

All visual impairments were more frequently seen after grade 4 IVHVD than after grade 3 (Figure 2); *p* < 0.05 (Fishers exact or χ^2^ test) for all except strabismus and the rectangles test. The median (IQR) number of impairments after grade 4 IVHVD was six (six to nine) compared with three (two to four) after grade 3 (Kolmogorov–Smirnov *p* = 0.003). Children who experienced grade 4 IVHVD were more likely to be in the top quartile for visual impairments (seven or more): OR = 6.43 (95% CI 1.03–40.26, *p* = 0.047).

After the same pre‐specified adjustments as above and adjusting for treatment arm, this association was not weakened but the confidence intervals widened even more: adjusted OR = 7.49 (95% CI 0.85–66.20, *p* = 0.070).

As shown in Figure 2, the excess of visual problems after grade 4 compared with grade 3 was particularly clear for the supranuclear eye movements, lag in accommodation, and the visuoperceptual/visuocognitive tests. All six children with a lag in accommodation had suffered grade 4 IVHVD.

Impaired eye movements or problems with the visuoperceptual/visuocognitive tests were more likely after grade 4 IVHVD than after grade 3: OR = 14.0 (95% CI 2.6–75.4, *p* = 0.002) for impaired pursuits; OR = 18.7 (95% CI 3.2–110.3, *p* = 0.001) for impaired saccades; and OR = 8.6 (95% CI 1.4–51.4, *p* = 0.019) for impairments in any one of the visuoperceptual/visuocognitive tests (postbox, contours, rectangles). There were also greater risks of having a strabismus (OR = 5.3 [95% CI 1.1–24.5, *p* = 0.030]) or reduced visual acuity (OR = 4.8 [95% CI 1.5–15.0, *p* = 0.008]) or impaired contrast sensitivity (OR = 5.1 [95% CI 1.0–28.7, *p* = 0.056]) after a grade 4 IVHVD compared with grade 3. Histograms of the eye movements scores after grade 3 or grade 4 IVHVD are shown in Figure S1.

### Associations between vision test results and BAS cognitive scores

Children who were able to do all the simple visuoperceptual/visuocognitive tasks had higher age‐adjusted centile scores for their spatial ability cluster of BAS tests than children who were unable to do one or more of these tasks. The median (IQR) spatial ability centile score was the 10th centile (3–42) compared with the 0.1 centile (0.1–2) for the children who could not do the simple tests (Kolmogorov–Smirnov *p* = 0.022).

Individual vision test results are compared with the BAS cluster scores in Table 2. Overall, the associations of vision problems with reduced cluster test scores were stronger for the spatial ability cluster (which depended on looking at the materials) than for the verbal ability cluster; however, there were also associations between some vision tests and the verbal ability cluster scores.

**TABLE 2:** Univariate comparisons between British Ability Scale, Third Edition scores converted to age‐standardized centiles and selected vision test results

Visual function/problem	Spearman's correlation coefficient with verbal ability cluster centile	*p*	Spearman's correlation coefficient with spatial ability cluster centile	*p*
Mean pursuit score	0.214	0.257	0.430	0.014
Mean saccade score	0.293	0.110	0.581	<0.001
Contrast sensitivity	0.250	0.174	0.473	0.006
Contour integration	−0.428	0.016	−0.588	<0.001
	**Median (IQR) verbal ability**	**Kolmogorov–Smirnov *p***	Median (IQR) spatial ability	Kolmogorov–Smirnov *p*
Reduced visual acuity				
No	17 (5–46)		14 (3.5–43.5)	<0.001
Yes	1 (0.1–2)	0.011	0.1 (0.1–0.4)	
Postbox				
Normal	12.5	0.052	7.5	0.008
Problem/could not do	0.1		0.1	
Rectangles				
Normal	17	0.009	10	0.001
Could not do	0.5		0.1	
Accommodation				
Normal	9	0.299	7	0.028
Lag	1		1	

Abbreviations: IQR, interquartile range; IVH, intraventricular haemorrhage; IVHVD, intraventricular haemorrhage and ventricular dilatation.

### Associations between vision test results and provision of extra help at school

The median (IQR) number of vision impairments for children with an SEN was seven (five to nine) compared with a median (IQR) of 2.5 (1.5–4) for children without an SEN (combined Kolmogorov–Smirnov *p* = 0.005). All children with reduced visual acuity, reduced peripheral visual field awareness, or nystagmus had SENs, as did all who could not do the postbox or rectangle matching tests. Children with either pursuit or saccade impairments, or reduced contour integration abilities, tended to be more likely to have an SEN, as seen in Table 3, including after adjustment for DAE, for eye movement control, and for number of impairments per child. However, adjustment by DAE must be interpreted with caution because the DAE was derived from a range of scores in the BAS cognitive tests, many of which were associated with the child's vision test results (Table 2).

**TABLE 3:** Associations between selected visual impairments and statement of special educational needs (SENs) or equivalent at age 10 years

Visual impairment	Unadjusted likelihood of having SEN OR (95% CI)	*p*	Adjusted for DAE OR (95% CI)	*p*
Impaired pursuits				
No (*n* = 16)	Reference	0.007	Reference	0.017
Yes (*n* = 16)	9.0 (1.8–44.6)		10.3 (1.5–70.0)	
Impaired saccades				
No (*n* = 17)	Reference	0.003	Reference	0.054
Yes (*n* = 15)	13.0 (2.4–70.5)		6.4 (1.0–41.7)	
Impaired (delta >1) contour integration skills				
No (*n* = 20)	Reference	0.068	Reference	0.262
Yes (*n* = 8)	5.6 (0.9–35.3)		3.4 (0.4–29.1)	
Lag in accommodation				
No (*n* = 20)	Reference		Reference	
Yes (*n* = 6)	5.6 (0.5–57.0)	0.147	2.2 (0.2–29.0)	0.558
Total number of visual impairments per child (out of 12)—increase per impairment	1.8 (1.2–2.7)	0.004	1.7 (1.1–2.6)	0.015

Abbreviations: CI, confidence interval; DAE, developmental age equivalent; OR, odds ratio.

### Additional analyses

The motion‐detection data were very sparse (*n* = 10) and normative data are yet to be collected with this specific set‐up. As motion detection has been reported as associated with stereoacuity^16^ we looked to see whether there was similar evidence within this cohort of children. There was an association between the motion scores and stereoacuity as median motion sensitivities were 25% congruently moving dots needed for the five children with stereoacuity of 800 seconds/arc or better versus 64% congruently moving dots needed by the five children with no or worse stereoacuity (combined Kolmogorov–Smirnov 1.00, *p* = 0.013).

## DISCUSSION

In this opportunistic, exploratory analysis we aimed to see whether there were differences in the visual outcomes of children who had been in the treatment or usual‐care arms of the study, or who experienced grade 4 IVHVD compared with grade 3 IVHVD, at 10 years of age. Although *p*‐values have been presented, the study was not sufficiently powered to answer these specific questions so the emphasis must be on the generation of hypotheses from the overall pattern of results. There was a trend for a greater number of visual problems after usual care compared with after the DRIFT treatment and consistently worse vision results after grade 4 IVHVD than after grade 3 IVHVD. This result was particularly marked for the supranuclear eye movements, accommodation, and visuoperceptual/visuocognitive tests (Figure 2). This may relate to the brain networks involved with these functions (superior longitudinal fasciculus, inferior longitudinal fasciculus, inferior fronto‐occipital fasciculus) being more easily damaged in grade 4 IVHVD, which involves blood extending into the periventricular areas through which the networks travel,^17^ whereas grade 3 IVHVD involves the blood being confined to the ventricles.

The high numbers of visual impairments found on direct testing are in contrast to the lower frequency of parent reports of visual difficulties affecting their children (25% in Table 1) and illustrates that parent‐reported visual outcomes may underestimate the true level of visual morbidity present in studies of infants and children. However, if the parents had been asked more detailed questions that related to the visual functions we tested (such as ocular alignment or contrast sensitivity), their responses may have shown more agreement with those of the direct assessment.

We saw multiple associations between impaired visual functions and impaired cognitive ability scores. Overall, these were more consistent and stronger for the vision‐dependent spatial ability scores than for the verbal ability scores. However, these participants were a highly selected patient group with multiple clinical problems, rather than a sample of the general population. Nevertheless, the children's performance in the simple clinical vision processing tasks (postbox, rectangles, contour integration) reflected the results of standard neuropsychology testing, supporting their use as simple indicators of visual processing abilities in children who have experienced similar clinical problems. Previous reports have associated saccadic function with various measures of IQ^18^ in adults and in children with CP,^19^ as reported here. What has not been reported before, to our knowledge, is the potentially strong association between impaired pursuits and/or saccades, with needing extra educational help (Table 3).

As visual function is important for information gathering, impaired visual function may well impair learning.^4^, ^20^, ^21^ Our results add to the evidence suggesting there is unmet visual need present for some children with additional needs.^22^ Specialist vision teachers usually are not involved with children unless they have reduced visual acuity or field, while our data suggest other visual impairments such as oculomotor or visuoperceptual/visuocognitive problems such as reduced contour integration skills may also impede learning.

This study is limited by the small number of self‐selected participants, who were more able than children who did not attend, so we may have underestimated the prevalence of vision problems. Some children may have seen better with up‐to‐date rather than their habitual glasses. Confrontation field testing is less accurate than formal perimetry and we may have missed some visual field defects as these have been reported in children after IVHVD.^23^ We did not include (for pragmatic reasons) all possible tests of visuoperceptual or visuocognitive functions, including some such as visual attention^24^ or object recognition that have been reported to show impairments affecting children who experienced IVHVD or other problems associated with preterm birth.^3^, ^25^, ^26^, ^27^ Therefore we are likely to have underestimated the number of visual impairments present in this cohort. We used the number of impairments per child as a proxy for their degree of visual morbidity, which does not capture the impact of the severity of individual visual defects; our results may have been different if we had included this aspect. We did not have access to the full ophthalmic records for all children and cannot comment on their previous diagnoses or management, or whether those differed according to the grade of IVHVD they experienced.

The study's strengths are that a carefully documented group of children with a specific diagnosis were followed prospectively and the examiners were masked to previous diagnosis and other data and used standardized assessments to describe a range of visual functions.

These results suggest clinicians and researchers should be aware of the high level of visual morbidity present at least 10 years after grade 3 or 4 IVHVD, especially grade 4. We hypothesize that developmental outcomes for children such as those presented here might be improved if support for their visual impairments can be instigated as early as possible. Our data suggest it would be useful to broaden the scope of vision assessments offered after IVHVD or other neurodevelopmental insults, to include tests such as those used here, as a guide to optimizing support strategies. Further research is needed to investigate this, with the aim of improving outcomes for these very vulnerable children.

## Funding information

CW was supported by a National Institute for Health and Care Research senior research fellowship. PW was supported by the Health Technology Assessment NIHR grant for the DRIFT10 study. The original DRIFT study was funded by the James and Grace Anderson Trust and by Cerebra.

## Supporting information


**Appendix S1:** Methods for vision testing in DRIFT10 follow‐up study and vision questionnaires used

**Figure S1:** Histograms showing pursuit and saccade movement scores for children with grade 3 vs grade 4 intraventricular haemorrhage

## Data Availability

Data available on request due to privacy/ethical restrictions
