# Supplementary material for: Vision function in children 10 years after grade 3 or 4 intraventricular haemorrhage with ventricular dilation: A masked prospective study
Source: Dev Med Child Neurol. 2022 Jun 23;65(2):223–31. doi: 10.1111/dmcn.15294 (PMC10084054; doi:10.1111/dmcn.15294)
Supplement: Supplementary file 1 — Appendix S1: Methods for vision testing in DRIFT10 follow‐up study and vision questionnaires used [file DMCN-65-223-s001.docx]

**Appendix S1**

**A.Methods for vision testing in DRIFT10 follow-up study**

**Visual Acuity** tested with both eyes open, using own glasses if worn. Test was ETDRS (Early treatment Diabetic Retinopathy Study) crowded LogMAR using a light box at 4m, or Kays Pictures crowded or single if unable to do LogMAR.

**Strabismus** tested using prism cover tests and uncover tests at 6 m and at 33 cm

**Contrast sensitivity** test was Cardiff Cards Contrast tests, impairment defined as worse than 66% (as per instructions with test)

**Peripheral visual awareness** tested using confrontation visual field test

**Accommodation** (near focussing) was assessed using dynamic retinoscopy and judged as accurate vs lag ie failing to accommodate on the target

**Eye movements (pursuits and saccades)** were graded on observation, ranging between 5=perfect to 1=unable to do, each tested horizontally and vertically, and pursuits tested for fast and slow target speed whilst saccades tested for small amplitude and large amplitude using standard target. Results of a small repeatability study included in Suppl materials of an earlier paper^1^

**Contour integration cards^2, 3^** (provided by Dr I Kovacs for a previous study) were used to assess contour integration and performance was defined as abnormal if delta was > 1.0 or greater, where delta was the ratio between the spacing of the distractor Gabor patches divided by the spacing of the target Gabor parches. If delta was >1, the contour was only detected when the contour Gabors were closer to each other than were the distractor Gabors, so the contour is made visible by the more dense spacing of the component Gabor patches

**Lea postbox task** was used and performance was defined as abnormal if a child couldn’t post the “letter” through the slit or had obvious difficulties, despite having adequate hand motor function

**Lea rectangles task** was used and performance was defined as abnormal if a child couldn’t copy either open or closed pattern, despite confirming that they could distinguish a “short wide” block from a “tall narrow block”

We used our locally collected, published norms to interpret the Lea tasks and the Contour Integration card results^4^.

**Stereoacuity** was measured using Randot preschool test

**B. Questions asked to Parents about their children’s vision**

1.In a general health questionnaire, they were asked:

How would you describe your child’s vision?

Normal

Normal with Correction

Useful but not fully correctable

Blind or can only perceive light

2. In the vision session they were asked questions asked based the Houliston et al Structured Inventory^5^. The responses to these questions have previously been presented ^6, 7^ and are not included in this paper, but are presented here as supplementary material for completeness

**Questions adapted from Houliston et al^5^**

| **Does your child have problems with:** | **1=Never** |  |  |  | **5=Always** |
| --- | --- | --- | --- | --- | --- |
| Parent recognition |  |  |  |  |  |
| Family recognition |  |  |  |  |  |
| Friend recognition |  |  |  |  |  |
| Photograph recognition |  |  |  |  |  |
| Self-photograph recognition |  |  |  |  |  |
| Shape recognition |  |  |  |  |  |
| Object recognition |  |  |  |  |  |
| Colour naming |  |  |  |  |  |
| Colour matching |  |  |  |  |  |
| Finding way in home |  |  |  |  |  |
| Asking way in home |  |  |  |  |  |
| Losing objects at home |  |  |  |  |  |
| Finding way in new places |  |  |  |  |  |
| Asking way in new places |  |  |  |  |  |
| Reaching and grasping objects |  |  |  |  |  |
| Distinguishing step from line |  |  |  |  |  |
| Misjudging doorways/corridoors |  |  |  |  |  |
| Can manage steps/kerbs |  |  |  |  |  |
| Seeing moving objects |  |  |  |  |  |
| Finding objects while moving |  |  |  |  |  |
| Finding objects in complex scenes/pictures |  |  |  |  |  |
| Finding objects pointed out in the distance |  |  |  |  |  |
| Eating food from part of the plate |  |  |  |  |  |

**References for supplementary materials**

1. Williams C, Pease A, Warnes P, Harrison S, Pilon F, Hyvarinen L*, et al.* Cerebral visual impairment-related vision problems in primary school children: a cross-sectional survey. *Dev Med Child Neurol*. 2021;63(6):683-9

2. Kovacs I, Polat U, Pennefather PM, Chandna A, Norcia AM. A new test of contour integration deficits in patients with a history of disrupted binocular experience during visual development. *Vision Res*. 2000;40(13):1775-83

3. Pennefather PM, Chandna A, Kovacs I, Polat U, Norcia AM. Contour detection threshold: repeatability and learning with 'contour cards'. *Spat Vis*. 1999;12(3):257-66

4. Williams C, Gilchrist ID, Fraser S, McCarthy HM, Parker J, Warnes P*, et al.* Normative data for three tests of visuocognitive function in primary school children: cross-sectional study. *Br J Ophthalmol*. 2015;99(6):752-6

5. Houliston MJ, Taguri AH, Dutton GN, Hajivassiliou C, Young DG. Evidence of cognitive visual problems in children with hydrocephalus: a structured clinical history-taking strategy. *Dev Med Child Neurol*. 1999;41(5):298-306

6. Luyt K, Jary S, Lea C, Young GJ, Odd D, Miller H*, et al.* Ten-year follow-up of a randomised trial of drainage, irrigation and fibrinolytic therapy (DRIFT) in infants with post-haemorrhagic ventricular dilatation. *Health Technol Assess*. 2019;23(4):1-116

7. Luyt K, Jary SL, Lea CL, Young GJ, Odd DE, Miller HE*, et al.* Drainage, irrigation and fibrinolytic therapy (DRIFT) for posthaemorrhagic ventricular dilatation: 10-year follow-up of a randomised controlled trial. *Arch Dis Child Fetal Neonatal Ed*. 2020;105(5):466-73
