# Supplementary material for: Vision function in children 10 years after grade 3 or 4 intraventricular haemorrhage with ventricular dilation: A masked prospective study
Source: Dev Med Child Neurol. 2022 Jun 23;65(2):223–31. doi: 10.1111/dmcn.15294 (PMC10084054; doi:10.1111/dmcn.15294)

Figure S1 Histograms showing (a) pursuit and (b) saccade movement scores for children with Grade 3 vs Grade 4 IVH

(a) Pursuit Eye Movement scores by grade of IVHVD


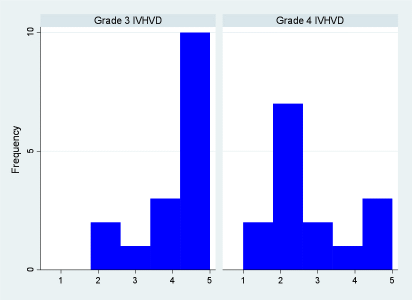


(b) Saccade Eye Movement Scores by grade of IVHVD


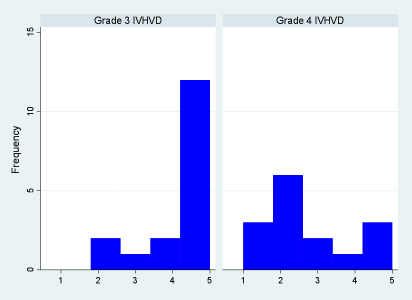

Supplement: Supplementary file 2 — Figure S1: Histograms showing pursuit and saccade movement scores for children with grade 3 vs grade 4 intraventricular haemorrhage [file DMCN-65-223-s002.docx]
